# Complete Genome Sequence of Paraburkholderia terrae Strain KU-15, a 2-Nitrobenzoate-Degrading Bacterium

**DOI:** 10.1128/mra.00373-22

**Published:** 2022-06-22

**Authors:** Yaxuan Liu, Kenji Okano, Hiroaki Iwaki

**Affiliations:** a Department of Life Science and Biotechnology, Kansai University, Suita, Osaka, Japan; Montana State University

## Abstract

Paraburkholderia terrae strain KU-15 has been investigated for its ability to degrade 2-nitrobenzoate. Here, we report the complete 10,422,345-bp genome of this microorganism, which consists of six circular replicons containing 9,483 protein-coding sequences. The genome carries genes that are potentially responsible for 2-nitrobenzoate and 4-nitirobenzoate degradation.

## ANNOUNCEMENT

Strain KU-15 was isolated as a 2-nitrobenzoate-degrading bacterium from agricultural soil and was also found to grow on 4-nitrobenzoate as the sole nitrogen and carbon source (1). 2-Nitrobenzoate is degraded by a unique pathway, via 2-hydroxylaminobenzoate branched to 3-hydroxyanthranilate and anthranilate. Strain KU-15 was tentatively identified as Paraburkholderia (formerly *Burkholderia*) terrae based on the 16S rRNA gene sequence (1). Members of the genus *Paraburkholderia* are known to degrade various aromatic and aliphatic compounds (2, 3), and members of the species P. terrae are known to be dinitrophenol and cyclohexanecarboxylate degraders (4, 5). Here, we report the complete genome sequence of P. terrae KU-15. This will expand our understanding of this potentially useful bacterial genus and species.

P. terrae KU-15 was obtained from our laboratory cryostock, and the genomic DNA was isolated from cells after 18 h of culture in 50 mL of one-half concentration of Miller’s LB medium (Merck Millipore) at 30°C by Wilson’s procedure (6), with some modifications. Cells were washed twice with Tris-EDTA buffer and then resuspended in 15 mL of the same buffer supplemented with 1 mg/mL RNase A; the amounts of the subsequent reagents were scaled up in relation to the volume of the cell suspension. For PacBio sequencing, 8 μg of the genomic DNA was sheared into fragments of the desired size (20 kb) using a g-TUBE (Covaris) and purified using AMPure PB magnetic beads (Beckman Coulter). A 20-kb SMRTbell template library was prepared and sequenced using an PacBio RS II instrument (Pacific Biosciences), and the subreads were filtered using PreAssembler Filter v1 (minimum subread and polymerase read lengths, 500 and 100 bp, respectively; minimum polymerase read quality, 0.80) in single-molecule real-time (SMRT) analysis v2.3.0 (Pacific Biosciences). In total, 114,407 reads, composed of 1,144,477,965 bp, with an *N*_50_ value of 14,180 bp, were obtained. The reads were *de novo* assembled using the FALCON-integrate protocol v2.1.4 (7). The assembled genome was 10,422,345 bp, consisting of six circular replicons. The genome sequence was annotated using DFAST (https://dfast.nig.ac.jp) (8). Default parameters were used for all tools unless otherwise noted. The basic genomic characteristics are shown in Table 1.

**TABLE 1:** General genomic features of Paraburkholderia terrae KU-15

Genome	Length (bp)	GC content (%)	Coverage (×)	No. of coding sequences	No. of rRNAs	No. of tRNAs	GenBank accession no.
Chromosome 1	3,743,440	62.64	85	3,305	12	56	AP025256
Chromosome 2	2,877,408	62.34	90	2,580	6	7	AP025257
Chromosome 3	2,290,229	61.98	91	2,038	0	2	AP025258
Chromosome 4	754,536	59.02	85	805	0	0	AP025259
Chromosome 5	692,026	59.76	86	685	3	2	AP025260
pPT65	64,706	59.64	72	70	0	2	AP025261
Total	10,422,345	61.94	87	9,483	21	69	

Strain KU-15 was identified using digital DNA-DNA hybridization (dDDH) (9, 10) and average nucleotide identity (ANI) (11) values. The dDDH value was calculated using the Type (Strain) Genome Server (TYGS) on the DSMZ website (http://tygs.dsmz.de) (12) with formula d4. The ANI values were calculated with JSpeciesWS (http://jspecies.ribohost.com/jspeciesws) using BLAST (13). The dDDH and ANI values in comparison with the type strain of P. terrae were 80.7% and 96.6%, respectively. According to the proposed criteria based on genomic data (14), strain KU-15 is a novel strain of the known species P. terrae, which is consistent with the results based on the 16S rRNA gene sequence.

To identify genes related to nitrobenzoate degradation, we performed BLAST searches using in silico Molecular Cloning software (in silico biology); the 2-nitrobenzoate-degrading genes (*nba*) of Pseudomonas sp. KU-7 (15) and the 4-nitrobenzoate-degrading genes (*pnb*) of Ralstonia pickettii YH105 (16) were used as query sequences. The *nba* genes, *nbaFCTAREHGIJDB* (locus tags PTKU15_72150 to PTKU15_72260), were located on chromosome 3, and genes encoding anthranilate dioxygenase subunits, *andAcAdAbAa* (PTKU15_72290 to PTKU15_72320), were located adjacent to the *nba* gene cluster. The *pnb* genes *pnbA* (PTKU15_35570), *pnbB* (PTKU15_35480), and *pnbR* (PTKU15_35500) were located on chromosome 2. This complete genome sequence offers a genetic basis for facilitating the elucidation of catabolic pathways for nitroaromatic compounds in this specialized bacterium and species P. terrae.

### Data availability.

The genome sequence of Paraburkholderia terrae strain KU-15 is available from DDBJ/EMBL/GenBank with accession numbers AP025256, AP025257, AP025258, AP025259, AP025260, and AP025261. The associated BioProject, BioSample, and Sequence Read Archive (SRA) accession numbers are PRJDB10639, SAMD00252780, and DRR322713, respectively.
